# Categorization of Indoor Places Using the Kinect Sensor

**DOI:** 10.3390/s120506695

**Published:** 2012-05-22

**Authors:** Oscar Martinez Mozos, Hitoshi Mizutani, Ryo Kurazume, Tsutomu Hasegawa

**Affiliations:** 1 Faculty of Information Science and Electrical Engineering, Kyushu University, 744 Motooka, Nishi-ku, Fukuoka 819-0395, Japan; E-Mails: kurazume@ait.kyushu-u.ac.jp (R.K.); hasegawa@ait.kyushu-u.ac.jp (T.H.); 2 Graduate School of Information Science and Electrical Engineering, Kyushu University, 744 Motooka, Nishi-ku, Fukuoka 819-0395, Japan; E-Mail: mizutani@irvs.ait.kyushu-u.ac.jp

**Keywords:** Kinect sensor, place categorization, service robots

## Abstract

The categorization of places in indoor environments is an important capability for service robots working and interacting with humans. In this paper we present a method to categorize different areas in indoor environments using a mobile robot equipped with a Kinect camera. Our approach transforms depth and grey scale images taken at each place into histograms of local binary patterns (LBPs) whose dimensionality is further reduced following a uniform criterion. The histograms are then combined into a single feature vector which is categorized using a supervised method. In this work we compare the performance of support vector machines and random forests as supervised classifiers. Finally, we apply our technique to distinguish five different place categories: corridors, laboratories, offices, kitchens, and study rooms. Experimental results show that we can categorize these places with high accuracy using our approach.

## 1. Introduction

An important capability for service robots working in indoor environments is their ability to categorize the different places where they are located. Place categorization has many applications in service robots. It is mainly used in semantic mapping, where acquired maps of the environment are extended with information about the type of each place allowing high level conceptual representations of environments [1–6]. In addition, the information about the type of a place can be used as prior or context information to improve the detection of objects [7, 8]. Moreover, whenever a robot has information about the type of a place, it can determine the possible actions to be carried out in that area [9–11].

In the task of place categorization a robot assigns a label to the place where it is located according to the information gathered with its sensors. The labels assigned by the robot to the different places are usually the same that people would use to refer to those places such as office, kitchen, or laboratory. In this way the communication with humans is improved [12, 13].

**Figure 1.**
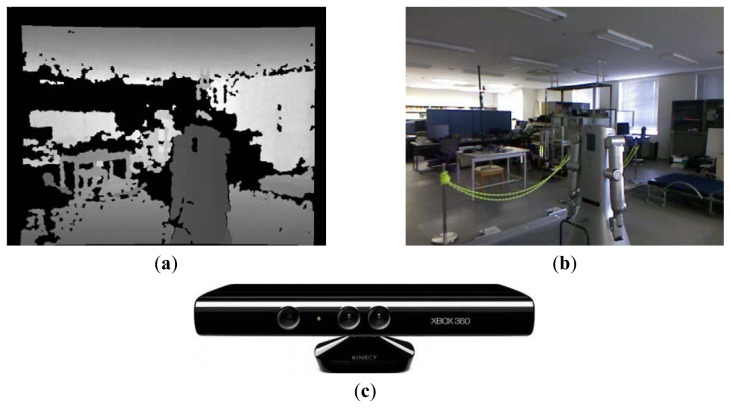
(**a**) Depth image obtained in a laboratory using the Kinect sensor. Different depths are shown using different grey values. Complete black pixels represent undefined values (see Section 3.2); (**b**) Corresponding RGB image representing the same scene; (**c**) The Kinect sensor used in our approach.

In this paper we present a new approach to categorize indoor places using a RGB-D sensor, in particular the Kinect camera [14]. The Kinect sensor is able to provide RGB and depth images simultaneously at high rates. Moreover, this sensor is getting popular in the robotics community due to its low cost. Figure 1 shows the Kinect sensor together with example depth and RGB images taken in a laboratory.

The main idea of our approach consists of transforming the image and depth information from the Kinect camera into feature vectors using histograms of local binary patterns (LBPs) whose dimensionality is reduced using a uniform criterion [15]. In order to obtain LBPs from RGB images they should first be transformed into grey scale images since the LBP operator ignores color information. The goal of this work is to distinguish categories of places, *i.e.*, places with similar structural and spatial properties, and for this reason we have selected a descriptor that does not take color properties into consideration. Previous works on place categorization [16, 17] also support the premise of ignoring color information for general categorization of indoor places.

The final feature vectors are combined and used as input to a supervised classifier. In this paper we compare the perform ance of support vector machines (SVMs) [18] and random forests (RFs) [19] as classification methods. We apply our method to sequences of images corresponding to five different place categories namely corridors, laboratories, offices, kitchens, and study rooms, and obtain average correct classification rates above 92%. This result demonstrates that it is possible to categorize indoor places using a Kinect sensor with high accuracy. Finally, we show the improvement of our categorization approach when using both modalities simultaneously (depth and grey images) in comparison with single modalities.

The rest of the paper is organized as follows: after presenting related work in Section 2, we introduce the local binary pattern transformation for grey scale and depth images in Section 3. In Section 4 we describe the combined feature vector used to represent the grey scale and depth images corresponding to the same scene. The supervised classifiers used for the categorization are presented in Section 5. We introduce our dataset in Section 6. Finally, experimental results are presented in Section 7.

## 2. Related Work

The problem of place recognition by mobile robots has gained much attention during recent years. Some previous works use 2D laser scans to represent different places in the environment. For example, in [20] 2D scans obtained with a laser range finder are transformed into feature vectors representing their geometrical properties. These feature vectors are categorized into several places using Boosting. The work in [21] uses similar feature vectors to represent locations in a Voronoi Random Field. Moreover, in [22] sub-maps from indoor environments are obtained by clustering feature vectors representing the different 2D laser scans. Finally, the work in [23] introduces the classification of a single scan into different semantic labels instead of assigning a single label to the whole scan.

Vision sensors have also been applied to categorize places indoors using mobile robots. In [16] the CENTRIST descriptor is applied to images representing different rooms in several houses. The descriptors are later classified using support vector machines. Moreover, in the PLISS system for place categorization introduced in [17] images are represented by bag of words using the SIFT descriptor. Similar images are grouped together by locating change-points in the sequences. In [7] local and global features from images taken by a wearable camera are classified using a hidden Markov model.

Finally, combinations of different modalities have been also applied to robot place recognition. The work in [24] combines 2D laser scans with visual object detection to categorize places indoors. Moreover, in [25] multiple visual and laser-based cues are combined using support vector machines for recognizing places indoors.

In contrast to these works, we use the new Kinect sensor which has the advantage of simultaneously providing visual and depth information. We apply a combination of image and depth images which allows us to integrate richer information about the visual appearance and the 3D structure of each place.

## 3. Local Binary Patterns

The local binary pattern (LBP) operator introduced in [15, 26] has been originally used for analysis and classification of grey scale images. The LBP is a local transformation that contains the relations between pixel values in a neighborhood of a reference pixel. In the next sections we explain how to calculate the LBP transformation for the RGB and depth images obtained with the Kinect sensor.

### 3.1. LPB Transformation for RGB Images

To apply the LBP transformation to RGB images they should be converted first into grey scale images because LBPs ignore color information and work only with intensity values. Then for each pixel *p_i_* in the grey scale image we calculate the corresponding LBP value following the approach presented in [15]. In particular, given a pixel *p_i_* with image coordinates (*x_i_, y_i_*), we compare its value *v*(*p_i_*) with the values corresponding to the 8-neighboring pixels *p_j_* ∈ *N_8_*(*p_i_*). For each neighboring pixel *p_j_* we obtain a binary value *b_j_* ∈ {0, 1} indicating whether the value *v*(*p_i_*) of the reference pixel *p_i_* is bigger than the value *v*(*p_j_*) of the neighboring pixel *p_j_* as:

(1) $$b_{j}=\{\begin{array}{lll} 1 & if & v(p_{i})>v(p_{j});\\ 0 & \text{otherwise}. & \end{array}$$

**Figure 2.**
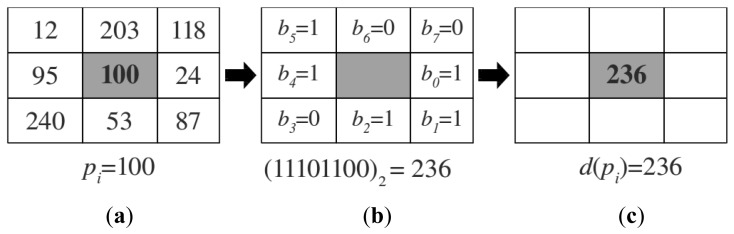
Toy example for the calculation of the LBP value of a pixel in a grey scale image. (**a**) The reference pixel *p_i_* (marked in bold in a shadow cell) has an initial value of 100; (**b**) Corresponding binary values for the 8-neighboring pixels of *p_i_*. The values are arranged into a binary string following a clockwise order starting at *b_0_* with a corresponding decimal value of 236; (**c**) The obtained decimal value is used as the new value for *p_i_* in the transformed image *T_grey_*.

**Figure 3.**
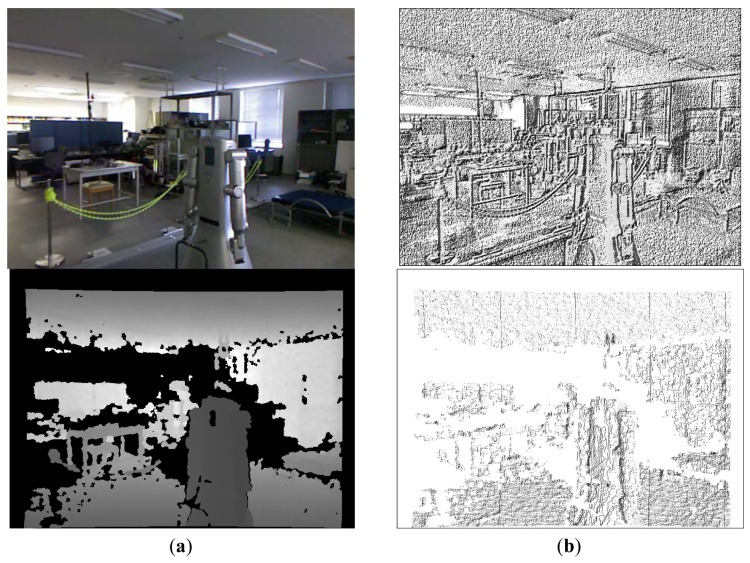
Example LBP transformations. (**a**) Original RGB (upper) and depth (bottom) images; (**b**) Corresponding LBP transformed images: *T_grey_* (upper) and *T_depth_* (bottom).

The binary values in the neighborhood are concatenated into a string in some specific order. In this work we use a clockwise order starting with the value *v*(*p_s_*) of the pixel which is on the right of the center pixel *p_i_*, that is, *p_s_* = (*x_i_* + 1, *p_y_*). The obtained binary string is then converted into the corresponding decimal value *d*(*p_i_*) ∈ [0, 255]. An example of this process is shown in Figure 2. The final LBP is obtained after applying the previous transformation to every pixel in the image, obtaining a final transformed image *T_grey_*. Figure 3 (upper row) shows the result of applying the LBP transformation to a RGB image obtained with the Kinect camera.

The abovementioned LBP operator is equivalent to the LBP_8,1_ operator of [15] with the solely difference that we do not interpolate values at the diagonals. Moreover, it is equivalent to the *Census Transform* presented in [27].

### 3.2. LPB Transformation for Depth Images

Pixels in depth images provided by the Kinect sensor represent the distance of objects to the sensor (see Figure 1(a)). To obtain the LBP transformation of depth images we apply the same process as for grey images (Section 3.1) but using the depth values. However, since the Kinect camera has a limited working depth range, the pixels representing depth values outside this range appear as undefined values in the corresponding depth image. In addition, we obtain similar undefined values when the camera is pointing to reflective surfaces, or when the pixels represent positions close to the borders of objects. Examples of these cases are presented in Figure 1(a) where undefined pixels are shown in black. To integrate undefined pixels when calculating the LBP transformation we propose to extend the range of resulting decimal values with the extra value 256 to represent these undefined cases. In addition, when calculating the LBP value for a given pixel in the depth image we also take into account neighboring pixels with undefined values as follows. For a given pixel *p_i_* in the original depth image we assign it the decimal value 256 if its depth value is undefined or there exists some undefined value in its 8-neighborhood *N_8_*(*p_i_*). Otherwise we apply the standard LBP procedure of Section 3.1. Formally:

(2) $$d^{+}(p_{i})=\{\begin{array}{lll} 256 & if & \delta (p_{i})\vee \forall p_{j}\in N_{8}(p_{i}),\exists \delta (p_{j})\\ d(p_{i}) & \text{otherwise}, & \end{array}$$

where *δ*(.) is an indicator function which returns *true* when its argument is an undefined value, and *false* otherwise. The value *d*(*p_i_*) is the base-10 value obtained by applying the LBP transformation of Section 3.1. Finally, the resulting value *d*^+^(*p_i_*) is contained in the extended range [0, 256]. After applying this operator to every depth pixel we obtain the resulting transformed image *T_depth_*. An example of a LBP transformation for a depth image is shown in Figure 3.

## 4. Multi-Modal Representation of Places

In our approach places are represented by depth and color images taken by a Kinect camera. In this section we explain how to combine both modalities to obtain a global feature vector which will be later categorized using different supervised methods.

The transformed images *T_grey_* and *T_depth_* obtained by following the steps of Section 3 are further represented by histograms *H_grey_* and *H_depth_* respectively. Each bin in these histograms contains the frequency of appearance of the different LBP transformed values. In the case of grey images the range of LBP transformed values *d*(*p_i_*) is [0, 255] and the corresponding histogram *H_grey_* contains 256 bins, one bin for each value. In the case of depth images the range of values *d*^+^(*p_i_*) is [0, 256] and the corresponding histogram *H_depth_* contains 257 bins (c.f. Section 3).

LBPs define local structures in images and histograms of LBPs represent the distribution in the scene of these local structures, and thus give a general representation of the images which in our case represent different place categories. Similar histograms may represent different places but these places should share a similar global structure. This is in fact an advantage in our approach since our objective is to classify places with similar global structure into the same category, e.g., different corridors should be include in the general category “corridor”, in the same way different offices should be detected as pertaining to the same category “office”. Histograms of local features have been successfully used in previous works to classify images into different place categories [16, 17, 28].

In our approach we further reduce the dimensionality of each histogram by selecting a subset of their LPBs using a *uniformity measurement U* introduced in [15] which indicates the number of transitions between 0/1 values of the binary representation of the decimal value *d* as:

(3) $$U(d)=\left|b_{0}-b_{7}\right|+\sum_{j=1}^{6}\left|b_{j}-b_{j+1}\right|,$$

where the different values b_i_ are obtained following Equation (1) and their position inside the image are indicated in Figure 2(b). As an example, the uniformity value corresponding to the decimal LBP value *d* = 236 is *U*(*d*) = 4 (c.f. Figure 2(b)).

As explained above LBPs represent local structure in the image (see Figure 2). Moreover, some of these local structures appear with different frequencies in different places, and also present different discriminative properties. In this paper we want to study the discriminative properties of these different local structures when they are applied to the problem of place categorization. For this purpose we use the uniformity measurement *U* to select different subsets of LBPs, *i.e.*, different local structures. In the experiments we will see that the selection of subsets of LBPs according to the uniformity measurement *U* improves the categorization results. A side effect of this selection is the reduction on the dimensionality in the final feature vectors representing different place categories; however, as the experiments will demonstrate, this reduction improves the classification results. We think this is due to the elimination of LBPs containing poor discrimination properties for place categorization. For example, when the threshold *θ* is high we allow LBPs corresponding to local structures with many local changes that can correspond to noise, while low thresholds maintain only more defined local structures like for example corners or lines as in Figure 2(b).

Using the uniformity measurement *U* the final histograms are composed of the subsets of bins representing the selected LBPs as:

(4) $$\begin{array}{c} H_{\text{grey}}^{\theta }=\{h_{d}|U(d)\leq \theta \},d\in [0,255]\\ H_{\text{depth}}^{\theta }=\{h_{d}|U(d)\leq \theta \}\cup \{h_{256}\},d\in [0,255] \end{array}$$

where *h_d_* is the bin in the histogram corresponding to LBP value *d*, and *θ* is a threshold for the uniformity measurement. Lower values for *θ* produce histograms with lower dimensionality. As an example, for *θ* = 2 the resulting histograms have 58 bins, while a value of *θ* = 4 results in histograms of 198 bins. When the threshold *θ* = 8 then there is no reduction in the corresponding histograms and they are equivalent to the CENTRIST descriptor, which has been recently introduced for place categorization using visual information [16]. That means that CENTRIS can be seen as a special case of our approach when *θ* = 8.

Finally, the multi-modal feature vector **x**^θ^ describing a particular place is obtained by concatenating the reduced histograms corresponding to both modalities:

(5) $${\text{x}}^{\theta }=\left\{H_{\text{grey}}^{\theta },H_{\text{depth}}^{\theta }\right\}$$

## 5. Classification

The multi-modal feature vector obtained in the previous section is used as input to a supervised method for categorization purposes. In this paper we compare two state-of-the-art classification methods: support vector machines, and random forests.

### 5.1. Support Vector Machines

The first supervised classification method is based on a support vector machine (SVM) [29, 30]. During the training phase, a support vector machine takes as input a set of *N* feature vectors **x***_i_* together with their binary labels *y_i_* ∈ {1, −1}. The idea behind SVMs is to find the hyperplane that maximizes the distance between the examples of the two classes. This is done by finding a solution to the optimization problem:

(6) $$\underset{w,b,\xi }{\min}C\sum_{i=1}^{N}\xi_{i}+\frac{1}{2}{\left\|w\right\|}^{2},$$

subject to the condition:

(7) $$y_{i}({\text{w}}^{T}\phi ({\text{x}}_{i})+b)\geq 1-\xi_{i},$$

where *w* is the normal to the hyperplane, and *ξ* ≥ 0 are slack variables that measure the error in the misclassification of **x***_i_*. In addition we use the radial basis function (RBF) kernel:

(8) $$K({\text{x}}_{i,}{\text{x}}_{j})=\phi {\left({\text{x}}_{i}\right)}^{T}\phi ({\text{x}}_{j})=\exp\left(-\gamma {\left\|{\text{x}}_{i}-{\text{x}}_{j}\right\|}^{2}\right),\gamma >0.$$

In the test step new examples **x**_t_ are labeled according to:

(9) $$y_{i}=({\text{w}}^{T}\phi ({\text{x}}_{t})+b).$$

SVMs were originally designed to solve binary classification problems. In the case of multi-class classification different approaches can be used to manage several classes. In our case we apply the “one-against-one” approach [31] which implies to learn a SVM for each pair of categories, resulting in a total of *k*(*k*-1)/2 classifiers for *k* categories.

In our experiments we use the implementation given by the LIBSVM library [32]. Moreover, the parameters *C* and *γ* are selected by grid-search using cross-validation in the ranges C ∈ [2^−5^, …, 2^15^] and *γ* ∈ [2^−12^, …, 2^3^] as described in [33]. Finally, the input feature vectors are first normalized in the range [0, 1].

### 5.2. Random Forests

The second type of supervised classifier used in this work is the random forest [19]. The idea behind this classifier is to use *M* classification trees each of which assigns a label to the input vector **x**. The final label is obtained by a majority vote over the labels assigned by all trees.

In this approach, each tree is trained as follows. First, using the original training data with *N* feature vectors, a new training set is created by random sampling of *N* samples with replacement. Second, during the creation of each node in the tree a subset of *l* ≪ *L* features from the total feature vector **x**∈ℝ*^L^* is randomly selected. Finally, the tree is constructed without pruning. In our approach we use the random forest implementation of WEKA [34] which is based on [19].

## 6. Place Dataset

**Figure 4.**
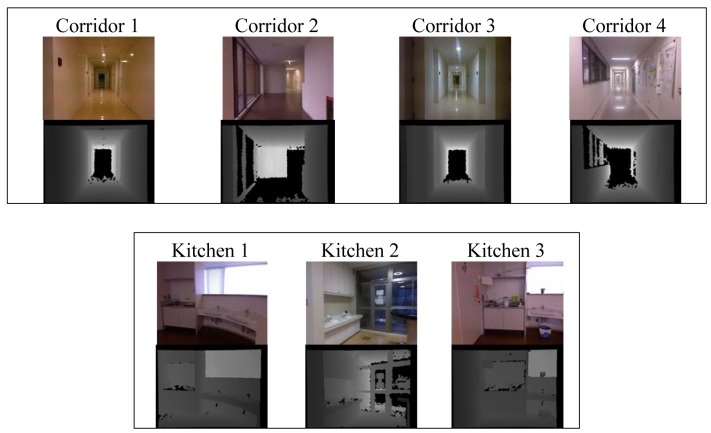

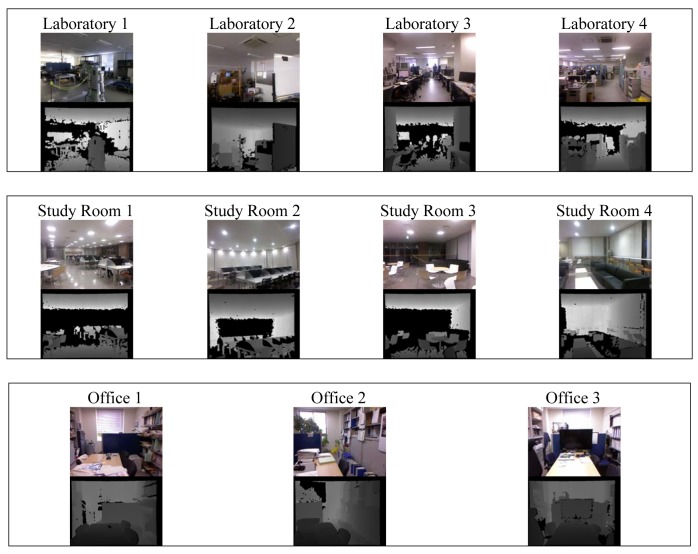
Examples of RGB and depth images for the places in each category.

To test our approach we have created a dataset of places by collecting data in different buildings at the University of Kyushu (this dataset is available at [35]). The dataset contains RGB and depth images acquired by a Kinect sensor which was mounted on a mobile platform at a height of 125 cm. We collected data from five different place categories: “corridor”, “kitchen”, “laboratory”, “office”, and “study room”. Each category contains RGB and depth images from several places that pertain to that category. For example the category “laboratory” contains data from four different laboratories. In each place we obtained one sequence of images while controlling the platform manually. The trajectory at each place has a different length and thus contains a different number of images. Table 1 presents a summary of the information contained in the dataset. For obtaining the place data we used the Robot Operating System framework (ROS) on a laptop equipped with an Intel core i5. In our experiments we simultaneously recorded depth images, 3D point clouds and RGB images. Since the Kinect camera does not provide hardware synchronization of RGB and depth images, we use the closest timestamp to match images of both modalities. The elapsed times between depth and RGB images ranged between 5 ms and 10 ms. Examples of RGB and depth images for each place in our dataset are shown in Figure 4.

## 7. Experiments

To evaluate the performance of our approach we conducted several experiments using our dataset of places. To create the different test and training sets for the experiments we applied the following procedure. Each test set was created by randomly selecting one place from each category, *i.e.*, each test set contains always five sequences of grey scale and depth images each of which corresponds to one category. Example test sets are {corridor 1, kitchen 2, laboratory 4, study room 1, office 2} or {corridor 2, kitchen 2, laboratory 3, study room 2, office 2}. The rest of places are used as training data. The idea behind this selection is that the test sets contain always sequences of places that do not appear in the training set, in this way we test the behavior of our method when applied to previously unseen places. Finally, for each experiment we repeated the previous process 10 times and obtained the average confusion matrices for the five categories.

We first show categorization results using our proposed approach in which we combined reduced histograms of LBP for grey scale and depth images that are classified using a SVM. In addition, we compare our approach with results in which the histograms of LBPs are not reduced.

Moreover, we show the improvement of the performance when using the combination of both modalities in comparison with single modalities only. We also present classification results applying *spatial pyramids* [28], a well known technique used in computer vision to improve classification results of scenes. Finally, we study the performance of our combined descriptor when used with support vector machines in comparison to random forests. In all the experiment the RGB images were first converted into grey scale.

### 7.1. Categorization of Places with Combined Histograms of LBP and SVMs

In the first experiment we study the performance of our approach when using histograms of reduced local binary patterns together with support vector machines. The final combined modality feature vectors **x** representing each pair of grey and depth images were obtained following the method of Section 4. In addition we apply different thresholds θ for the uniformity measurement and compare their classification results. As explained above, we repeated 10 experiments using different training and test sets. The support vector machines for each of the 10 experiments were trained using RBF kernels whose parameters were found by grid-search (see Section 5.1).

Table 2 presents the overall classification results for the 10 experiments. Results are averaged over the 10 experiments and are accompanied by the corresponding standard deviations. As Table 2 suggests best results are obtained with threshold θ = 4. In this case not only the average classification rate improves but also the uncertainty (represented by the standard deviation) is reduced. When θ = 8 there is no reduction in the histograms of LBPs and the final descriptor is equivalent to CENTRIST [16].

**Figure 5.**
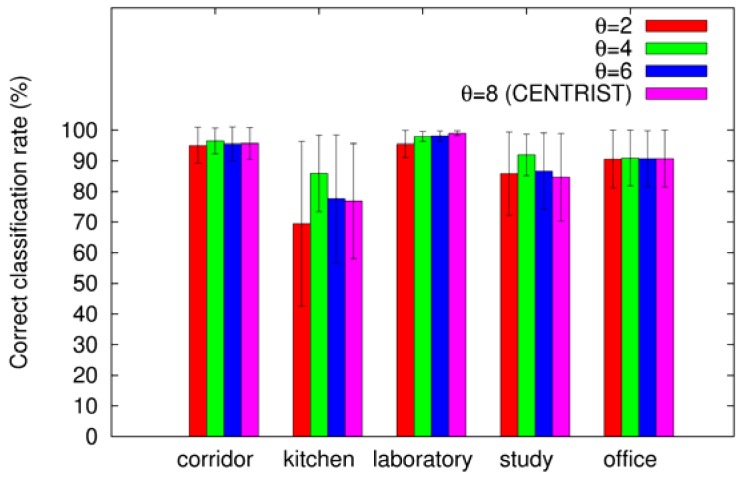
Correct classification rates by category using different uniformity thresholds.

In addition, Figure 5 plots the average correct classification rates for each category. As shown in the plot best results are obtained almost always when θ = 4. In particular, the performance greatly improves in the most difficult categories which are “kitchen” and “study room”.

Finally, we present the details of the previous experiments using confusion matrices which indicate the predicted classification for the actual place. The value of each cell in the confusion matrix is the average and standard deviation over the 10 experiments. The confusion matrices for different values of the uniformity threshold θ are shown in Table 3.

### 7.2. Multiple Modalities vs. Single Modalities

**Figure 6.**
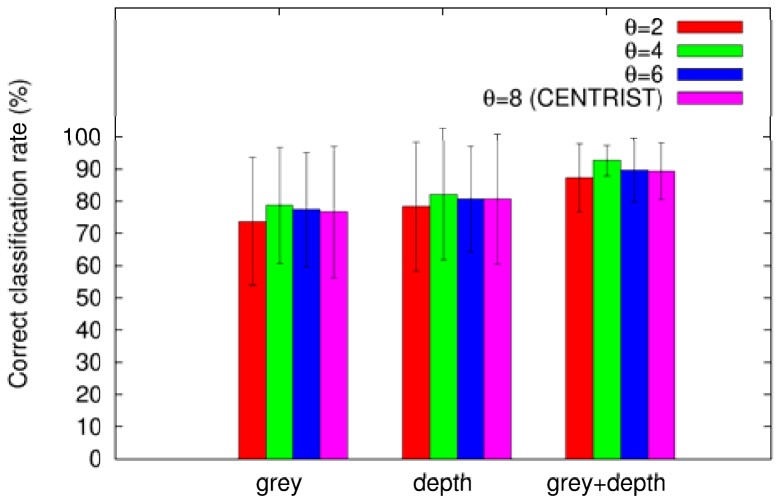
Correct classification rates using different modalities.

In this section we study the improvement on the categorization of places when using the combined modalities (grey and depth images) in comparison with single modalities only (grey or depth image). We repeated the experiments of the previous section using different data each time: grey images only, depth images only, and grey + depth images. Similar to the previous section we used SVMs as classifiers. Figure 6 compares the overall categorizations using different uniformity thresholds for each modality. As we can conclude from the plot, the combination of grey and depth images outperforms the categorization using single modalities only. We also can appreciate that combining the modalities the uncertainty (represented by the error bars) is drastically reduced. Moreover, in all modalities the reduced histograms using θ = 4 perform best.

Another conclusion that can be obtained from these results is that categorization using only depth information is slightly better than the categorization using grey images only. This can be due to the fact that depth information encodes general structures of indoor places and it is invariant to changes in illumination.

### 7.3. Categorization Using Spatial Pyramids

**Figure 7.**
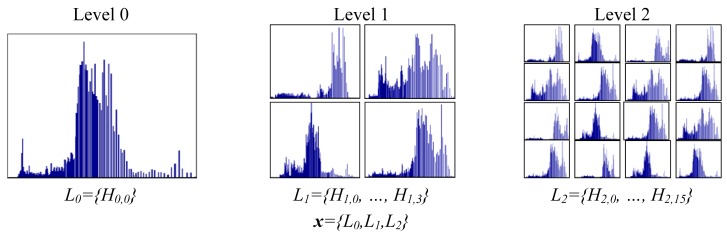
Histograms using Spatial Pyramids [28]. Three levels of pyramids are applied and the corresponding local histograms are concatenate to form the final feature vector **x**.

In this section we study the performance of our categorization system when applying spatial pyramids [28]. Spatial pyramids is a well known technique that is used to capture the structure of an image at different locations. The idea behind a spatial pyramid is to divide the original image into different parts. Each local part is treated as an individual image and their respective histogram is calculated. This process is applied at different levels. The final feature vector is obtained by concatenating the local histograms from all levels. A graphical example of this technique is given in Figure 7. At each level *i* we generate *2^i^* × *2^i^* histograms. The final feature vector **x** is obtained by concatenating the histograms of all levels.

We applied spatial pyramids using the data from our previous 10 experiments using SVM as classifiers and compare different modalities and uniformity thresholds. A final summary of categorization results is shown in Table 4 showing overall average correct categorization results and standard deviation for the 10 experiments. The results in Table 4 show that the combination of modalities outperforms single ones in almost all cases. We also can see that the best result in the combined modality is obtained in level 0. Previous literature reported better results when applying spatial pyramids to image categorization. From Table 4 we can see that this is also the case when using individual modalities, *i.e.*, grey scale images or depth images only, however the combination of both does not improve the categorization at further levels in our particular dataset and experiments. We want to study this behavior in future work.

### 7.4. Classification Using Random Forests

In this section we compare the performance of our approach when using random forests in the categorization step. We compare the performance with the best results obtained using SVMs with reduced feature vectors using uniform measurement threshold θ = 4. Table 5 shows a summary of this comparison. As we can see the use of support vector machines outperforms random forest at different levels of spatial pyramids. In this table we can also see that results using random forest improve as the levels of spatial pyramids increase; however we do not observe this behavior when using the multi-class implementation of SVM provided in libsvm [32].

## 8. Conclusions

In this paper we have presented a method to classify places in indoor environments using RGB and depth images obtained by a Kinect camera. Our approach uses a combination of both modalities to create a feature vector that is categorized using different supervised methods. Moreover, we have introduced the uniform measurement to reduce the combined feature vectors and to improve the final categorization results. In addition, we compared the categorization results using SVMs and random forests. The results indicated that SVMs are more appropriate for our particular case. Finally, the results in all our experiments demonstrated that the combination of depth and image information outperforms the use of single modalities individually.

In this work, we did not apply any extra reduction of dimensionality in the final combined feature vectors used for categorization. However, when using spatial pyramids at different levels the dimension of the feature vectors grows exponentially and the application of some reduction technique such as PCA can improve results at subsequent levels [16]. As future work we want to study different methods to further reduce the dimensionality of the feature vectors at different levels and compare these results to the ones presented in this paper. We also want to study new ways of combining vectors from different modalities.

## Figures and Tables

**Table 1.** Dataset containing a total of 1,228 pairs of RGB and depth images.

**Category**	**Place**	**RGB and depth images**

Corridor	Corridor 1	68
	Corridor 2	42
	Corridor 3	70
	Corridor 4	99

	Total	279

Kitchen	Kitchen 1	73
	Kitchen 2	65
	Kitchen 3	53

	Total	191

Laboratory	Laboratory 1	99
	Laboratory 2	99
	Laboratory 3	81
	Laboratory 4	78

	Total	357

Study Room	Study Room 1	71
	Study Room 2	70
	Study Room 3	49
	Study Room 4	62

	Total	252

Office	Office 1	57
	Office 2	45
	Office 3	47

	Total	149

**Table 2.** Overall classification results using SVMs and different uniformity thresholds. We show the average and standard deviations over 10 experiments.

θ = 2	θ = 4	θ = 6	θ = 8 (CENTRIST)
87.27 ± 10.71	**92.61** ± **4.78**	89.71 ± 9.92	89.37 ± 8.85

**Table 3.** Confusion matrices for place categorization using SVMs and different uniformity thresholds.

**θ** = **2**		Predicted Class				

	%	Corridor	Kitchen	Laboratory	Study room	Office
	
Actual class	Corridor	**95.05** ± **7.02**	0.20 ± 0.63	3.84 ± 6.25	0.91 ± 1.93	0.00 ± 0.00
	Kitchen	2.64 ± 3.99	**69.43** ± **30.68**	4.15±7.96	22.64 ± 25.32	1.13 ± 2.97
	Laboratory	0.25 ± 0.78	1.24 ± 3.90	**95.51** ± **6.45**	2.26 ± 2.97	0.75 ± 1.94
	Study Room	0.00 ± 0.00	3.29 ± 4.61	10.57 ± 11.15	**85.82** ± **14.66**	0.32 ± 1.02
	Office	0.00 ± 0.00	4.39 ± 4.69	5.09 ± 5.57	0.00 ± 0.00	**90.53** ± **10.10**

**θ** = **4**		Predicted Class				

	%	Corridor	Kitchen	Laboratory	Study room	Office
	
Actual class	Corridor	**96.47** ± **5.15**	0.91 ± 1.53	2.02 ± 4.04	0.61 ± 1.59	0.00 ± 0.00
	Kitchen	2.64 ± 2.38	**85.88** ± **14.04**	1.51 ± 2.48	7.89 ± 9.83	2.08 ± 2.87
	Laboratory	0.00 ± 0.00	0.20 ± 0.42	**97.91** ± **2.49**	0.77 ± 0.89	1.12 ± 2.69
	Study Room	0.00 ± 0.00	5.14 ± 5.47	2.29 ± 3.37	**91.93** ± **8.00**	0.65 ± 2.03
	Office	0.00 ± 0.00	3.51 ± 4.60	5.61 ± 6.65	0.00 ± 0.00	**90.88** ± **9.63**

**θ** = **6**		Predicted Class				

	%	Corridor	Kitchen	Laboratory	Study room	Office
	
Actual class	Corridor	**95.53** ± **5.51**	2.23 ± 2.85	2.23 ± 3.39	0.00 ± 0.00	0.00 ± 0.00
	Kitchen	2.57 ± 2.25	**77.62** ± **20.82**	2.07 ± 2.94	14.51 ± 16.30	3.21 ± 3.80
	Laboratory	0.00 ± 0.00	0.22 ± 0.44	**98.08** ± **1.64**	0.94 ± 0.65	0.75± 1.46
	Study Room	0.00 ± 0.00	8.13 ± 9.03	4.89 ± 4.64	**86.64** ± **12.47**	0.32 ± 0.95
	Office	0.00 ± 0.00	3.86 ± 4.42	5.45 ± 5.80	0.00 ± 0.00	**90.68** ± **9.15**

**θ** = **8 (CENTRIST)**		Predicted Class				

	%	Corridor	Kitchen	Laboratory	Study room	Office
	
Actual class	Corridor	**95.66** ± **5.92**	2.02 ± 2.65	2.32 ± 3.81	0.00 ± 0.00	0.00 ± 0.00
	Kitchen	2.45 ± 2.36	**76.85** ± **21.45**	3.21 ± 4.79	12.40 ± 14.50	5.10 ± 5.18
	Laboratory	0.00 ± 0.00	0.12 ± 0.38	**99.02** ± **1.13**	0.23 ± 0.48	0.63 ± 0.88
	Study Room	0.00 ± 0.00	8.86 ± 11.81	5.57 ± 6.78	**84.64** ± **18.04**	0.93 ± 2.13
	Office	0.00 ± 0.00	3.51 ± 4.60	5.79 ± 6.77	0.00 ± 0.00	**90.70** ± **9.85**

**Table 4.** Comparison of single and combined modalities. Results are shown as percentages together with standard deviations.

		Grey	Depth	Grey + Depth

θ = 2	Level 0	73.72 ± 19.84	78.37 ± 20.03	87.27 ± 10.71
	Level 1	80.93 ± 21.79	83.22 ± 16.40	85.53 ± 19.46
	Level 2	82.21 ± 23.26	84.93 ± 17.18	82.46 ± 23.67

θ = 4	Level 0	78.75 ± 18.01	82.15 ± 20.53	92.61 ± 4.78
	Level 1	78.56 ± 23.13	89.02 ± 10.77	88.10 ± 15.75
	Level 2	78.87 ± 22.80	86.67 ± 16.28	88.95 ± 14.18

θ = 6	Level 0	77.38 ± 17.73	80.70 ± 16.40	89.71 ± 9.92
	Level 1	80.33 ± 17.44	85.08 ± 12.58	87.18 ± 12.4
	Level 2	78.33 ± 18.18	82.18 ± 15.55	80.69 ± 15.32

θ = 8 (CENTRIST)	Level 0	76.60 ± 20.43	80.72 ± 20.14	89.37 ± 8.85
	Level 1	79.47 ± 21.78	85.11 ± 17.52	85.68 ± 17.88
	Level 2	82.18 ± 18.30	83.14 ± 20.13	84.59 ± 19.69

**Table 5.** Comparison of SVM and random forest as categorization methods using as input reduced feature vectors with uniform measurement threshold θ = 4. Results are shown in percentages.

Level	SVM	Random Forest

0	92.61 ± 4.78	85.74 ± 11.82
1	88.10 ± 15.76	87.57 ± 14.23
2	88.95 ± 14.18	88.43 ± 12.79
